# Improvement of muscle quality in tilapia (*Oreochromis niloticus*) with dietary faba bean (*Vicia faba* L.)

**DOI:** 10.3389/fnut.2023.1153323

**Published:** 2023-03-27

**Authors:** Qingqing Li, Yao Huang, Xingqian Zhang, Cuiyun Zou, Li Lin

**Affiliations:** ^1^Guangdong Provincial Water Environment and Aquatic Products Security Engineering Technology Research Center, Guangzhou Key Laboratory of Aquatic Animal Diseases and Waterfowl Breeding, College of Animal Sciences and Technology, Zhongkai University of Agriculture and Engineering, Guangzhou, Guangdong, China; ^2^Guangdong Zhongshan Tilapia Science and Technology Backyard, Ministry of Education, Guangzhou, Guangdong, China

**Keywords:** faba bean, texture, muscle fiber, amino acid, fatty acid, *Oreochromis niloticus*

## Abstract

Tilapia (*Oreochromis niloticus*) is a freshwater fish which is farmed worldwide. Improving the muscle quality of fish has become a major goal while maintaining a sustainable aquaculture system. This research attempts to assess the effect of 0% (FB0), 40%(FB40), 50%(FB50), 60%(FB60) and 70% (FB70) faba bean on the texture parameter, histological analysis, proximate, amino acids, and fatty acids composition in tilapia fed 90 days. The results showed that hardness, chewiness, and shear force of tilapia muscle fed FB60, and FB70 were considerably more in comparison to fish fed FB0 at 90 days (*p* < 0.05). Tilapia fed faba beans had higher muscle fiber density, wider spaces between muscle fibers and smaller fiber diameter, with the greatest difference in tilapia fed FB60. The total protein content in tilapia fed FB40 was considerably more in comparison to in fish fed FB70 (*p* < 0.05), where the total protein content in muscle first increased and then reduced with increasing dietary faba bean level. The muscle ∑TAA, ∑EAA, valine, tyrosine, cysteine, aspartic acid, methionine, isoleucine, glutamic acid, leucine, arginine, and serine, contents in tilapia fed FB60 were much more in contrast to in fish fed FB0 (*p* < 0.05), which initially increased and then reduced with increasing dietary faba bean level. The muscle ∑PUFA content in tilapia fed dietary faba beans was greater compared with fish fed FB0, whereas the ∑SFA contents in tilapia fed FB50 and FB60 were lower in contrast to in fish fed FB0. In summary, dietary faba beans can improve muscle texture, muscle fibers, amino acids content and fatty acids content in tilapia. The research’s results make a contribution to the improved knowledge of the association among muscle quality in tilapia and dietary faba beans.

## 1. Introduction

Tilapia (*Oreochromis niloticus*) is the second most common farmed fin fish group all over the world after carp (1), and its worldwide aquaculture production increased from 2.6 M tons in 2010 to 6.9 M tons in 2020 (2). This production is due to its excellent characteristics, such as rapid growth, high reproduction, resistance to high stocking densities, and high marketability (1, 3). Fish welfare may be impacted owing to increased stress brought on by growing production under intensive culture conditions, which will also have an impact on fish quality (4). The muscle is the most interesting part of the fish body in terms of aquaculture products. Therefore, to improve muscle quality, more sustainable aquaculture needs to be formed, necessitating better technology and management (5).

The term “fish muscle quality” refers to a group of qualities including freshness, texture, wholesomeness, nutritional content, and integrity. The sensory interpretation and representation of a product’s structure or inner construction, as well as its haptic qualities and reaction to stress, are all related to its texture (6). An essential aspect of fish muscle’s freshness is its texture, which is influenced by a number of factors including its cohesiveness, hardness, chewiness, springiness, adhesiveness, and resilience (7). Along the fish value chain, textural criteria are widely used to measure fish quality, which primarily entails consideration of how processing as well as handling techniques affect fish products’ shelf life as well as consumer preferences and satisfaction (7). Moreover, the quality of muscle is significantly impacted by composition and nutritional value (8). The fatty acid, protein, and amino acid content of fish represents its primary nutritional value (9). The precursors of a number of flavors are amino acids, which could alter the taste indirectly through the Maillard reaction, especially odor and flavor compounds. Through the process of oxidation, which also forms volatile molecules, lipids have a vital part in the cooked flesh of fish. Hence, the aquaculture industry’s main problem is how to produce high-quality cultured fish.

Both internal and external elements, such as culture environment (5, 8), food nutrition (10, 11), and genetics (12) have an impact on the quality of fish muscle. Fish flesh quality can be improved through nutrient control, which has been shown to be beneficial (13). Fish muscle quality improvement defined as higher muscle hardness and crispiness by feeding faba bean (*Vicia faba* L.), has gained increasing research interest (14–17). Crisped fish is very popular in China and other countries due to its special flesh quality. A previous study reported that the chewiness, flesh hardness, adhesiveness, and elasticity of grass carp (*Ctenopharyngodon idella*) were significantly increased by feeding the fish faba beans (18). Faba bean supplementation obviously influenced muscle fatty acid composition in the grass carp (19). Therefore, faba beans may be an effective measure for improving fish muscle quality. Understanding the impact of faba beans on skeletal muscle quality is important for optimizing feed used for high-quality tilapia production.

This study aims to assess the impact of various dietary proportions of faba beans on the muscle texture, muscle histology, and nutritional composition of tilapia. The results will contribute to an understanding of the muscle quality affecting the tilapia crisping process.

## 2. Materials and methods

### 2.1. Experimental tilapias and diets

A 90-day rearing trial was conducted at the Laboratory of Aquaculture situated at Zhongkai University of Agriculture and Engineering. Faba bean was added to the diets at 0% (FB0, the control group) and 40% (FB40), 50% (FB50), 60% (FB60), and 70% (FB70) as shown in Table 1. When the faba beans content was increased in the diets, the soybean, rapeseed, wheat flour, and soybean oil contents were progressively decreased to maintain the crude protein (31.73% ~ 32.76%) and lipid content (7.52% ~ 7.60%). The dry ingredients of experimental diets were mixed, and oil and water (25% v/w) were added to the dry mixture to form a soft dough, then the mixed dough was extruded and air-dried.

**Table 1:** Ingredients and proximate composition of experimental diets (%, air dry basis).

Ingredients (% dry matter)	Diet 1	Diet 2	Diet 3	Diet 4	Diet 5
Fish meal	10.00	10.00	10.00	10.00	10.00
Soybean meal	25.30	13.70	10.80	7.90	5.00
Rapeseed meal	26.00	14.00	11.00	8.00	5.00
Wheat flour	29.00	13.00	9.00	5.00	1.00
Soybean oil	3.20	2.80	2.70	2.60	2.50
Fish oil	2.50	2.50	2.50	2.50	2.50
Ca(H_2_PO_4_)2•H_2_O	1.00	1.00	1.00	1.00	1.00
Vitamin premix	1.50	1.50	1.50	1.50	1.50
Mineral premix	1.50	1.50	1.50	1.50	1.50
Faba bean meal	0.00	40.00	50.00	60.00	70.00
Total	100.00	100.00	100.00	100.00	100.00
Proximate composition					
Crude protein	32.76	32.17	32.03	31.88	31.73
Crude lipid	7.52	7.57	7.58	7.59	7.60
Ash	6.53	6.56	6.81	7.07	7.32
Moisture	6.12	6.11	6.13	6.12	6.14

The 300 tilapias (average initial body weight: 463.86 ± 16.51 g) were randomly and evenly assigned to the above five experimental diets, and triplicate for each experimental diet. The fish were cultured in 15 PVC tank (Φ108 × H120 cm) indoor circulating systems with a density of 20 tilapia/tank. A temperature of 28°C-29°C, dissolved oxygen ≥5 mg/l, pH 7.5, a light intensity of 100 lx and a photoperiod of 14 h in lightness and 10 h in darkness were maintained. The daily feeding amount were given ratios of around 3% of total fish biomass twice a day at 9:00 and 16:00, adjusted in accordance with satiation and residual feed.

### 2.2. Sample collection

Tilapia was monitored monthly until harvested at 90 days. The survival rate of tilapia in all groups has no significant difference and were greater than 90%. Every 30 days, three tilapias were randomly obtained from each tank. At each sampling point, the fish fasted for 24 h. Fish that were chosen at random were anesthetized rapidly in a 10 l plastic bucket with 20 mg/l clove oil (20). Each tilapia’s body weight was measured employing a digital balance and recorded to the nearest 0.01 g. Tilapias were sacrificed by decapitation, peeled and dissected employing sterile scissors from the anus to the throat on ice, followed by removal of visceral mass. The 3 cm × 3 cm × 5 cm muscle samples were isolated for filet texture profile analysis (TPA), and the muscle filet (0.5 cm × 0.5 cm × 0.5 cm) at the junction of the fourth dorsal fin and lateral line scales was sampled with the aid of a scalpel blade, washed in phosphate-buffered saline (PBS) and fixed in 2.5 percent glutaraldehyde for 2 h at room temperature, and then transferred to 4°C for preservation and transportation for transmission electron microscopy (TEM). The remaining muscle samples were swiftly separated, immediately frozen in liquid nitrogen, and then immediately kept at −80°C. Three muscle samples per group at days were dried used a vacuum freeze-dryer, ground to a fine powder with a mortar and pestle, then stored at −80°C until the proximate, fatty acid, and amino acid composition were determined.

### 2.3. Texture profile analysis

Texture profile analysis (TPA) of raw tilapia filets was carried out as explained by (21) with certain modifications. Three sampling points for TPA with a volume of 1 cm^3^ in duplicate were chosen between the dorsal fin and tail above the lateral line. TPA was carried out utilizing a texture analyzer (TA-X plus, Stable Micro Systems, United Kingdom) fitted out an 8 mm cylinder probe. The muscle textural parameters (chewiness, firmness, resilience, springiness, shear force, and cohesiveness) were determined. At an initial force of 0.1 N and a constant speed of 30 mm/min, two sequent compression cycles were conducted to make sure that the original length of the muscle sample reached 60% deformation.

### 2.4. Transmission electron microscopy

The tissue blocks were washed with 0.1 M PB (pH 7.4) three times, for a total of 15 min, after the samples were sliced into 1 mm × 1 mm × 2 mm pieces. The tissue blocks were then placed for one to 2 h at room temperature in 1 percent OsO4 in 0.1 M PB (pH 7.4). The tissue blocks were then washed three times in 0.1 M PB (pH 7.4) for 15 min each, followed by 20 min in 30 percent ethanol, 20 min in 50 percent ethanol, 20 min in 70 percent ethanol, 20 min in 80 percent ethanol, and 20 min in 95 percent ethanol. Two washes with 100 percent ethanol for 20 min each were then performed, and then isoamyl acetate for 15 min. The following actions were then taken: Acetone:EMBed 812 = 1:1 for 2–4 h at 37°C; Acetone:EMBed 812 = 1:2 overnight at 37°C; pure EMBed 812 for 5–8 h at 37°C; The pure EMBed 812 was poured into the embedding models and the tissues inserted into the pure EMBed 812, and then left in an oven at 37°C overnight. The embedding models with resin and samples were placed in an oven at 65°C to polymerize for over 48 h. After being taken out of the embedding models, the resin blocks were set aside to cool. The resin blocks were cut into 60–80 nm sections utilizing an ultra-microtome, and the tissues were placed onto 150 mesh cuprum grids with formvar film. 2 percent uranium acetate saturated alcohol solution avoid light staining for 8 min, rinsed in 70 percent ethanol 3 times and then rinsed in ultra-pure water 3 times. 2.6 percent lead citrate avoids CO^2^ staining for 8 min, followed by rinsing with ultra-pure water 3 times. The cuprum grids were placed in the grids board and allowed to dry overnight at room temperature after being dried with filter paper. The cuprum grids were observed under TEM (Hitachi, HT7800) and the images were recorded.

### 2.5. Proximate composition analysis

The fish muscle samples in each treatment group at 90 days was analyzed for moisture content (drying samples in a 105°C oven until constant weight), total lipid content (extracted with chloroform: methanol (2:1, v/v)), and total protein content (Kjeldahl method, utilizing 6.25 N as a protein conversion factor) in accordance with the technique of AOAC (22).

### 2.6. Amino acid analysis

Each muscle sample (about 0.2 g dry weight) was placed in a 10 ml hydrolysis tube. 10 ml of 6.0 M HCl solution was then added. The samples were left at −20°C for 5 min, filled with nitrogen and sealed, then hydrolyzed in a 110°C drying box for 22 h. The samples were transferred into hydrolysis solution in a 50 ml volumetric flask, rinsed with deionized water several times and the washing solution was pooled. After hydrolysis, 2 ml of the hydrolysate was taken out and evaporated at 60°C under vacuum to dryness in order to eliminate the HCl. In 5 ml of 0.02 N HCl, the hydrolysate was dissolved before being centrifuged used 2,237 x *g*, and then filtered through a 0.45 μm syringe filter. 1 μl of supernatant was employed for amino acid analysis, by means of pre-column orthophthalaldehyde and the 9-formic acid methyl ester of fluorine chlorine derivatization. The tryptophan content was analyzed separately, and pre-weighted samples were hydrolyzed at 5 M NaOH (containing 5% SnCl_2_, w/v) in 110°C for 20 h. The hydrolysate was neutralized with 6 M HCl following hydrolysis, centrifuged used 2,659 x *g*, and filtered through syringe filter. The retention periods and peak areas of standard amino acids (AAS18-5ML, DCH (Shanghai) Co., Ltd. Shanghai, China) were compared to the identity and quantity of the amino acids. The amino acid contents of muscle were expressed as individual amino acids in gram per 100 grams of dry muscle (g/100 g dry weight).

### 2.7. Fatty acid analysis

According to Morrison and Smith (23) approach, fatty acid methyl esters (FAME) were created by transesterification with boiling 15% borontrifluoride/methanol (w/w). After injecting the sample into a Thermo trace 1,310 gas chromatograph equipped with an Agilent CP-sil88 fused silica capillary column (length 100.0 m, inner diameter 0.25 mm, film thickness 0.2 μm), the fatty acid content was analytically confirmed utilizing flame ionization detection (FID). The temperature of the injector and detector was maintained at 270°C. The column temperature was first set at 100°C for 13 min, 10°C/min to 180°C for 6 min, 1°C/min to 200°C for 20 min, and 2°C/min to 220°C for 6 min until all FAME were eluted. Helium was employed as the carrier gas, and the flow velocity was 40 ml/min. Individual fatty acids were quantified by making reference to the internal standard, and FAME in the sample was recognized by comparing retention durations with known standards. Fatty acid contents of muscle were expressed as individual fatty acids in milligrams per gram dry muscle (mg/g dry weight).

### 2.8. Statistical analysis

The mean ± standard error (SE) of the data was computed utilizing SPSS software (version 20.0). Duncan’s multiple-range test and one-way analysis of variance (ANOVA) were employed for checking for the considerable disparity between the treatment groups at the same time points. The linear and quadratic effects of increasing the dietary faba bean level were performed through orthogonal polynomial contrast at the same time points. Differences of *p* < 0.05 were deemed statistically significant.

## 3. Results

### 3.1. Texture parameters

The influence of various dietary faba bean levels on the texture parameters were presented in Table 2. The hardness of tilapia muscle fed FB40, FB50, FB60 and FB70 was considerably greater in comparison to that in fish fed FB0 at 30 days (*p* < 0.05), however, no substantial disparity exists in hardness between fish fed FB40, FB50, FB60, and FB70. The hardness of tilapia muscle fed FB60 and FB70 was substantially higher than that in fish fed FB0 at 60 days and 90 days (*p* < 0.05), however, no substantial disparity exists in hardness between fish fed FB0, FB40 and FB50. The chewiness of tilapia fed FB60 and FB70 was substantially higher than that in fish fed FB0 at 60 and 90 days (*p* < 0.05), however, no substantial disparity exists hardness between fish fed FB50, FB60, and FB70. The shear force of tilapia fed FB60 and FB70 was considerably greater in comparison to that in fish fed FB0 at 90 days (*p* < 0.05), but there was no substantially disparity at 30 and 60 days. No substantial impact of dietary faba beans exists on the springiness, cohesiveness, and resilience of tilapia.

**Table 2:** Effects of dietary broad bean levels on texture profile analysis in muscle of tilapia *Oreochromis niloticus.*

Items	FB0	FB40	FB50	FB60	FB70	Linear		Quadratic
						*P*	*R* ^2^	*P*	*R* ^2^
30 days										
Hardness	647.75 ± 6.45^b^	911.90 ± 41.11^a^	976.30 ± 18.10^a^	1011.65 ± 39.58^a^	994.17 ± 10.35^a^	0.000	0.834	0.000		0.891
Springiness	0.62 ± 0.01	0.60 ± 0.03	0.59 ± 0.01	0.66 ± 0.02	0.62 ± 0.02	0.343	0.100	0.450		0.181
Chewiness	355.04 ± 93.42	405.47 ± 96.32	391.31 ± 30.37	448.46 ± 26.11	418.71 ± 42.38	0.009	0.549	0.037		0.561
Cohesiveness	0.66 ± 0.01	0.59 ± 0.04	0.62 ± 0.02	0.62 ± 0.02	0.62 ± 0.02	0.306	0.116	0.414		0.198
Resilience	0.32 ± 0.01	0.30 ± 0.02	0.32 ± 0.01	0.30 ± 0.01	0.31 ± 0.01	0.672	0.021	0.767		0.064
Shear force	969.30 ± 129.15	1038.73 ± 194.24	1043.57 ± 53.61	950.73 ± 143.10	954.63 ± 14.34	0.807	0.007	0.956		0.011
60 days										
Hardness	688.13 ± 97.62^b^	940.03 ± 124.04^ab^	1039.40 ± 182.59^ab^	1244.97 ± 218.20^a^	1176.60 ± 87.34^a^	0.027	0.401	0.098		0.403
Springiness	0.65 ± 0.03	0.68 ± 0.01	0.63 ± 0.01	0.66 ± 0.01	0.65 ± 0.01	0.020	0.436	0.062		0.461
Chewiness	308.80 ± 68.75^b^	400.64 ± 51.78^ab^	421.01 ± 25.23^ab^	460.62 ± 17.14^a^	456.76 ± 21.40^a^	0.010	0.500	0.044		0.501
Cohesiveness	0.68 ± 0.02	0.66 ± 0.02	0.65 ± 0.01	0.64 ± 0.01	0.69 ± 0.02	0.643	0.022	0.377		0.195
Resilience	0.32 ± 0.02	0.30 ± 0.01	0.31 ± 0.02	0.32 ± 0.02	0.30 ± 0.01	0.555	0.036	0.698		0.077
Shear force	930.93 ± 58.56	1107.27 ± 90.84	1127.15 ± 26.95	1169.47 ± 111.17	1176.30 ± 129.04	0.342	0.091	0.652		0.091
90 days										
Hardness	707.27 ± 89.75^b^	1097.87 ± 133.92^ab^	1108.10 ± 170.80^ab^	1393.00 ± 52.34^a^	1329.43 ± 101.31^a^	0.007	0.503	0.028		0.511
Springiness	0.66 ± 0.03	0.61 ± 0.03	0.64 ± 0.01	0.63 ± 0.02	0.65 ± 0.02	0.560	0.032	0.614		0.093
Chewiness	327.03 ± 58.67^b^	541.47 ± 75.45^b^	427.80 ± 64.96^ab^	586.21 ± 32.90^a^	550.36 ± 38.71^a^	0.008	0.482	0.033		0.494
Cohesiveness	0.67 ± 0.02	0.63 ± 0.01	0.65 ± 0.02	0.65 ± 0.01	0.65 ± 0.02	0.152	0.177	0.366		0.182
Resilience	0.31 ± 0.01	0.31 ± 0.01	0.32 ± 0.01	0.32 ± 0.01	0.32 ± 0.01	0.004	0.540	0.016		0.654
Shear force	791.17 ± 95.17^b^	1171.88 ± 184.93^ab^	1202.18 ± 117.64^ab^	1355.03 ± 186.91^a^	1274.60 ± 69.03^a^	0.030	0.360	0.105		0.363

Data are represented as mean ± SE (*n* = 9). Using one-way ANOVA, compared with the control, data at the same elapsed time with different letters are significantly different (*P* < 0.05) among treatments.

### 3.2. Myofibrillar microstructure

**Figure 1:**
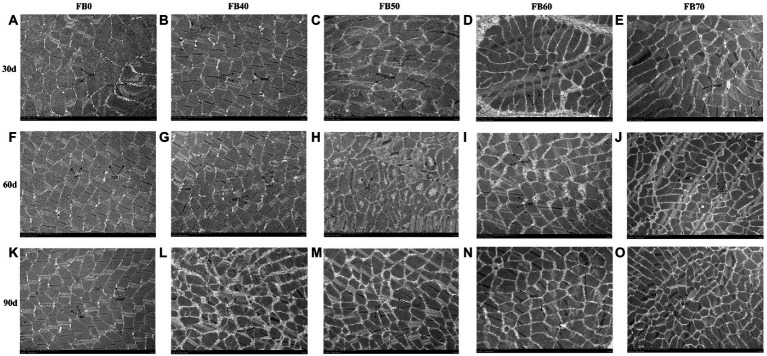
Effects of dietary faba bean levels on muscle transverse section microstructure by transmission electron microscope of tilapia *Oreochromis niloticus*. **(A)**, Control group on 30 day **(D)**; **(B)**, 40% faba bean group on 30 d; **(C)**, 50% faba bean group on 30 d; **(D)**, 60% faba bean group on 30 d; **(E)**, 70% faba bean group on 30 d; **(F)**, control group on 60 d; **(G)**, 40% faba bean group on 60 d; **(H)**, 50% faba bean group on 60 d; **(I)**, 60% faba bean group on 60 d; **(J)**, 70% faba bean group on 60 d; **(K)**, control group on 90 d; **(L)**, 40% faba bean group on 90 d; **(M)**, 50% faba bean group on 90 d; **(N)**, 60% faba bean group on 90 d; **(O)**, 70% faba bean group on 90 d. mf, muscle fiber; mmf, matrix between muscle fibers.

The muscle TEM images of tilapia fed dietary faba bean were shown in Figure 1. The muscle fiber diameter significantly decreased with dietary faba bean in contrast to the control group, while muscle fiber density enhanced substantially with dietary faba bean, especially in fish fed FB60 and FB70. Wider intermyofibrillar spaces were also seen in muscle samples of fish fed faba beans comapred to the control group.

### 3.3. Proximate composition

The total protein content in tilapia fed FB40 was considerably greater in comparison to that in fish fed FB70 (*p* < 0.05), however, no substantial disparity exists in total protein content between fish fed FB50, FB60, and FB70. The total lipid content in tilapia fed FB40 was substantially less than that in fish fed FB0 (*p* < 0.05), however, there was no substantial disparity in total lipid content between fish fed FB40, FB50, FB60, and FB70. No considerable impact of dietary faba beans exists on the moisture of tilapia (Table 3). The total protein content in muscle initially enhanced followed by reduction with enhancing dietary faba bean level, while the total lipid content indicated the opposite trend.

**Table 3:** Effects of dietary faba bean levels on proximate composition (% dry weight) in muscle of tilapia *Oreochromis niloticus.*

Items	FB0	FB40	FB50	FB60	FB70	Linear		Quadratic
						*P*	*R* ^2^		*P*	*R* ^2^
Moisture	74.85 ± 0.19	75.88 ± 0.27	75.69 ± 0.53	76.19 ± 1.00	75.89 ± 0.18	0.155	0.109	0.226	0.161	
Protein	77.78 ± 0.92^ab^	80.05 ± 0.56^a^	78.38 ± 0.33^ab^	78.50 ± 0.54^ab^	77.58 ± 1.00^b^	0.878	0.001	0.010	0.416	
Lipid	3.88 ± 0.45^a^	2.37 ± 0.34^b^	2.68 ± 0.41^ab^	2.67 ± 0.66^ab^	2.67 ± 0.35^ab^	0.222	0.082	0.015	0.392	

Data are represented as mean ± SE (*n* = 3). Data with different letters are significantly different in the same tissues (*P* < 0.05) between treatments, and the no letters are not significantly different.

### 3.4. Amino acid composition

The muscle ∑TAA content in tilapia fed FB60 was considerably greater in comparison to that in fish fed FB0 (*p* < 0.05), however, no considerable disparity exists in ∑TAA content between fish fed FB40, FB50, FB60, and FB70. The muscle ∑EAA content in tilapia fed FB60 was considerably greater in comparison to that in fish fed FB0 and FB70 (*p* < 0.05), however, no substantial disparity exists in hardness between fish fed FB40, FB50, and FB60. The ∑TAA and ∑EAA contents in tilapia first increased followed by reduction with increasing dietary faba bean level. Regarding valine, essential amino acids, methionine, cysteine, arginine, isoleucine, and leucine contents in tilapia fed FB60 were considerably higher in comparison to those in fish fed FB0 (*p* < 0.05), which first increased followed by reducing with increasing dietary faba bean level. Regarding aspartic acid, non-essential amino acids, serine, and tyrosine contents, and glutamic acid in tilapia fed FB60 were considerably higher in comparison to those in fish fed FB0 (*p* < 0.05), while no substantial impact of dietary faba beans exists on glycine, alanine, and proline contents in tilapia (Table 4).

**Table 4:** Effects of dietary faba bean levels on amino acid composition (g/100 g dry weight) in muscle of tilapia *Oreochromis niloticus.*

Items	FB0	FB40	FB50	FB60	FB70	Linear		Quadratic
						*P*	*R* ^2^		*P*	*R* ^2^
Threonine	3.18 ± 0.03^c^	3.33 ± 0.04^ab^	3.37 ± 0.05^a^	3.34 ± 0.07^ab^	3.20 ± 0.05^bc^	0.879	0.001	0.002	0.386	
Valine	3.37 ± 0.03^b^	3.57 ± 0.04^a^	3.52 ± 0.07^ab^	3.60 ± 0.07^a^	3.45 ± 0.05^ab^	0.412	0.026	0.027	0.250	
Cystine	0.37 ± 0.02^b^	0.40 ± 0.01^b^	0.41 ± 0.01^b^	0.48 ± 0.03^a^	0.36 ± 0.02^b^	0.635	0.009	0.036	0.233	
Methionine	2.27 ± 0.05^b^	2.38 ± 0.06^ab^	2.41 ± 0.03^ab^	2.49 ± 0.08^a^	2.30 ± 0.06^b^	0.514	0.017	0.042	0.223	
Isoleucine	3.38 ± 0.04^b^	3.55 ± 0.04^ab^	3.50 ± 0.06^ab^	3.68 ± 0.13^a^	3.46 ± 0.06^b^	0.292	0.043	0.070	0.191	
Leucine	5.49 ± 0.05^c^	5.83 ± 0.08^ab^	5.76 ± 0.10^ab^	5.97 ± 0.20^a^	5.59 ± 0.10^bc^	0.480	0.019	0.024	0.259	
Histidine	1.78 ± 0.01	1.75 ± 0.05	1.81 ± 0.07	1.77 ± 0.03	1.70 ± 0.04	0.332	0.036	0.358	0.079	
Arginine	4.31 ± 0.04^b^	4.51 ± 0.05^ab^	4.51 ± 0.07^ab^	4.54 ± 0.10^a^	4.40 ± 0.08^ab^	0.448	0.022	0.036	0.234	
Phenylalanine	3.32 ± 0.04^ab^	3.31 ± 0.05^ab^	3.29 ± 0.01^ab^	3.37 ± 0.07^a^	3.21 ± 0.06^b^	0.229	0.055	0.322	0.087	
Lysine	6.51 ± 0.07	6.79 ± 0.08	6.70 ± 0.12	6.82 ± 0.13	6.59 ± 0.11	0.627	0.009	0.112	0.161	
∑EAA	33.96 ± 0.31^b^	35.41 ± 0.34^ab^	35.27 ± 0.58^ab^	36.05 ± 0.88^a^	34.25 ± 0.60^b^	0.645	0.008	0.032	0.240	
Aspartic acid	6.75 ± 0.08^b^	7.21 ± 0.08^a^	7.00 ± 0.09^ab^	7.09 ± 0.12^a^	6.82 ± 0.12^ab^	0.853	0.001	0.030	0.245	
Glutamic acid	10.49 ± 0.06^b^	11.26 ± 0.16^a^	11.05 ± 0.19^a^	11.09 ± 0.20^a^	10.76 ± 0.18^ab^	0.609	0.010	0.012	0.297	
Glycine	3.08 ± 0.04	3.23 ± 0.07	3.24 ± 0.05	3.22 ± 0.06	3.19 ± 0.11	0.372	0.031	0.226	0.112	
Alanine	4.11 ± 0.05	4.27 ± 0.07	4.23 ± 0.07	4.25 ± 0.09	4.11 ± 0.06	0.830	0.002	0.104	0.166	
Serine	2.81 ± 0.03^c^	2.97 ± 0.04^a^	3.04 ± 0.04^a^	2.96 ± 0.05^ab^	2.84 ± 0.05^bc^	0.897	0.001	0.000	0.487	
Tyrosine	2.74 ± 0.03^b^	2.82 ± 0.04^ab^	2.84 ± 0.02^ab^	2.89 ± 0.07^a^	2.81 ± 0.07^ab^	0.168	0.072	0.089	0.176	
Proline	2.27 ± 0.03	2.29 ± 0.08	2.28 ± 0.05	2.17 ± 0.01	2.16 ± 0.09	0.106	0.097	0.212	0.117	
Tryptophan	0.42 ± 0.01^a^	0.42 ± 0.01^a^	0.35 ± 0.01^b^	0.35 ± 0.00^b^	0.32 ± 0.00^c^	0.000	0.731	0.000	0.732	
∑NEAA	32.67 ± 0.25^b^	34.39 ± 0.44^a^	34.03 ± 0.48^ab^	34.00 ± 0.62^ab^	33.00 ± 0.55^ab^	0.928	0.000	0.022	0.264	
∑TAA	66.63 ± 0.55^b^	69.79 ± 0.77^ab^	69.29 ± 1.05^ab^	70.06 ± 1.50^a^	67.25 ± 1.13^ab^	0.772	0.003	0.024	0.258	

Data are represented as mean ± SE (*n* = 3). Data with different letters are significantly different in the same tissues (*P* < 0.05) between treatments, and the no letters are not significantly different. ∑EAA: essential amino acids; ∑NEAA: non-essential amino acids; ∑TAA: total amino acids.

### 3.5. Fatty acid composition

The impact of different dietary faba bean levels on the fatty acid contents were presented in Table 5. The muscle ∑SFA and ∑MUFA contents in tilapia fed dietary faba bean (FB40, FB50, FB60, and FB70) were slightly less than those in fish fed FB0, however, not significantly different. The C14:0 and C24:0 contents in fish fed FB40 were considerably lower in contrast to in fish fed FB0 (*p* < 0.05). The ∑MUFA content in muscle was generally not much impacted by dietary faba bean level, except that C16:1 and C18:1n9t decreased with increased dietary faba bean level. The ∑PUFA contents in tilapia fed FB50 and FB60 were slightly more in contrast to those in fish fed FB0, but not substantially different.

**Table 5:** Effects of dietary broad bean levels on fatty acid composition (mg/g dry weight) in muscle of tilapia *Oreochromis niloticus.*

Items	FB0	FB40	FB50	FB60	FB70	Linear		Quadratic
						*P*	*R* ^2^		*P*	*R* ^2^
C4:0	0.01 ± 0.00	0.01 ± 0.00	0.02 ± 0.00	0.01 ± 0.00	0.01 ± 0.00	0.605	0.021	0.756	0.046	
C12:0	0.10 ± 0.01	0.04 ± 0.01	0.05 ± 0.02	0.06 ± 0.020	0.06 ± 0.02	0.178	0.135	0.078	0.346	
C13:0	0.01 ± 0.00	0.00 ± 0.00	0.01 ± 0.01	0.01 ± 0.00	0.01 ± 0.00	0.278	0.090	0.430	0.131	
C14:0	0.70 ± 0.13^a^	0.22 ± 0.07^b^	0.32 ± 0.13^ab^	0.42 ± 0.13^ab^	0.36 ± 0.11^ab^	0.064	0.239	0.034	0.430	
C15:0	0.07 ± 0.01	0.06 ± 0.01	0.10 ± 0.03	0.09 ± 0.01	0.09 ± 0.01	0.331	0.073	0.592	0.084	
C16:0	7.32 ± 0.86	4.65 ± 0.72	6.00 ± 1.40	6.60 ± 1.12	5.75 ± 1.05	0.397	0.056	0.396	0.143	
C17:0	0.07 ± 0.00	0.07 ± 0.01	0.13 ± 0.04	0.07 ± 0.04	0.11 ± 0.01	0.378	0.060	0.689	0.060	
C18:0	2.53 ± 0.18	2.27 ± 0.19	2.96 ± 0.45	2.75 ± 0.04	2.57 ± 0.16	0.554	0.028	0.844	0.028	
C20:0	0.07 ± 0.00	0.07 ± 0.01	0.12 ± 0.04	0.10 ± 0.02	0.09 ± 0.01	0.172	0.139	0.393	0.144	
C21:0	0.01 ± 0.00	0.01 ± 0.00	0.02 ± 0.01	0.02 ± 0.01	0.01 ± 0.00	0.557	0.027	0.847	0.027	
C22:0	0.03 ± 0.00	0.03 ± 0.01	0.04 ± 0.02	0.04 ± 0.01	0.03 ± 0.01	0.418	0.051	0.724	0.052	
C24:0	0.16 ± 0.01^a^	0.10 ± 0.01^b^	0.12 ± 0.02^ab^	0.12 ± 0.01^ab^	0.13 ± 0.01^ab^	0.087	0.208	0.026	0.456	
∑SFA	11.07 ± 1.17	7.53 ± 1.04	9.90 ± 2.15	10.29 ± 1.32	9.23 ± 1.40	0.512	0.034	0.507	0.107	
C14:1	0.03 ± 0.00^a^	0.00 ± 0.00^b^	0.00 ± 0.00^b^	0.01 ± 0.01^b^	0.01 ± 0.00^b^	0.055	0.255	0.005	0.593	
C16:1	0.98 ± 0.22^a^	0.25 ± 0.09^b^	0.38 ± 0.16^b^	0.55 ± 0.21^b^	0.43 ± 0.14^b^	0.048	0.268	0.029	0.447	
C18:1n9t	0.07 ± 0.01^a^	0.03 ± 0.01^b^	0.04 ± 0.01^b^	0.05 ± 0.01^b^	0.03 ± 0.01^b^	0.035	0.300	0.034	0.430	
C18:1n9c	6.92 ± 1.35	3.94 ± 1.07	5.66 ± 1.98	6.50 ± 1.61	5.15 ± 1.21	0.540	0.030	0.599	0.082	
C20:1	0.33 ± 0.06	0.17 ± 0.04	0.29 ± 0.10	0.29 ± 0.06	0.22 ± 0.04	0.372	0.062	0.521	0.103	
C22:1n9	0.02 ± 0.00	0.01 ± 0.01	0.02 ± 0.01	0.02 ± 0.01	0.02 ± 0.00	0.658	0.016	0.902	0.017	
C24:1	0.01 ± 0.00	0.00 ± 0.00	0.01 ± 0.01	0.01 ± 0.00	0.01 ± 0.00	0.277	0.090	0.382	0.148	
∑MUFA	8.36 ± 1.65	4.41 ± 1.22	6.41 ± 2.26	7.43 ± 1.91	5.88 ± 1.41	0.424	0.050	0.476	0.116	
C18:2n6c	4.51 ± 0.43	4.80 ± 0.89	6.38 ± 1.83	6.74 ± 1.34	5.78 ± 1.22	0.223	0.112	0.490	0.112	
C20:2	0.27 ± 0.03	0.27 ± 0.14	0.56 ± 0.12	0.54 ± 0.03	0.42 ± 0.04	0.073	0.226	0.213	0.227	
C22:2	0.01 ± 0.00	0.01 ± 0.01	0.01 ± 0.01	0.02 ± 0.00	0.01 ± 0.00	0.343	0.069	0.406	0.139	
C18:3n3	0.35 ± 0.03	0.26 ± 0.08	0.37 ± 0.15	0.41 ± 0.11	0.36 ± 0.10	0.733	0.009	0.830	0.031	
C18:3n6	0.22 ± 0.05	0.15 ± 0.05	0.17 ± 0.05	0.22 ± 0.04	0.17 ± 0.05	0.623	0.019	0.705	0.056	
C20:3n3	0.07 ± 0.01	0.08 ± 0.01	0.12 ± 0.04	0.12 ± 0.01	0.10 ± 0.01	0.139	0.160	0.342	0.164	
C20:3n6	0.40 ± 0.03	0.27 ± 0.14	0.45 ± 0.06	0.51 ± 0.02	0.37 ± 0.05	0.673	0.014	0.815	0.033	
C20:4n6	1.53 ± 0.06	1.44 ± 0.04	1.54 ± 0.08	1.52 ± 0.02	1.42 ± 0.15	0.520	0.033	0.803	0.036	
C22:6n3	1.66 ± 0.15^ab^	1.48 ± 0.08^b^	2.16 ± 0.35^a^	1.84 ± 0.04^ab^	1.93 ± 0.20^ab^	0.264	0.095	0.511	0.106	
∑PUFA	9.02 ± 0.62	8.75 ± 1.32	11.77 ± 2.63	11.92 ± 1.50	10.56 ± 1.54	0.246	0.102	0.524	0.102	
∑HUFA	3.20 ± 0.17^ab^	2.92 ± 0.12^b^	3.70 ± 0.39^a^	3.37 ± 0.06^ab^	3.35 ± 0.22^ab^	0.429	0.049	0.715	0.054	
∑n-3PUFA	2.08 ± 0.20	1.81 ± 0.17	2.65 ± 0.54	2.37 ± 0.16	2.38 ± 0.31	0.294	0.084	0.536	0.099	
∑n-6PUFA	2.15 ± 0.13	1.86 ± 0.19	2.16 ± 0.17	2.26 ± 0.05	1.96 ± 0.25	0.828	0.004	0.930	0.012	
n-6/n-3	1.05 ± 0.12	1.03 ± 0.09	0.87 ± 0.15	0.95 ± 0.01	0.82 ± 0.10	0.140	0.159	0.298	0.183	

Data are represented as mean ± SE (*n* = 3). Data with different letters are significantly different in the same tissues (*P* < 0.05) between treatments, and the no letters are not significantly different. The fatty acids content less than 0.1 mg/g dry weight were not in the table. The fatty acids composition of less than 0.01 were not shown in the table. ΣSFA: total amount of saturated fatty acids; ΣMUFA: total amount of monounsaturated fatty acids; ΣPUFA: total amount of polyunsaturated fatty acids; ΣHUFA: highly unsaturated fatty acid; Σn-3PUFA: total amount of *n*-3 polyunsaturated fatty acids; Σn-6PUFA: total amount of n-6 polyunsaturated fatty acids; *n*-6/*n*-3: *n*-6/*n*-3 polyunsaturated fatty acids ratio.

## 4. Discussion

Nutritional content, texture, and appearance are the crucial quality factors that must be determined in fish muscle. The “crisp taste” is the distinctive characteristic of crisp fish, and certain investigations have found indices reflecting this trait (17). Textural parameters (chewiness, hardness, and springiness) have been linked to the “crisp taste” (18, 24, 25). The mechanical processing of filets and acceptance are both impacted by texture, a crucial quality attribute. Textural features, hardness especially, are linked to the intrinsic structure as well as the properties of components of the flesh and are closely related to the human fish products’ visible acceptance (7). The hardness of tilapia fed FB60 and FB70 was considerably higher in contrast to that in fish fed FB0. The increased hardness of muscle because of feeding on faba bean has likewise been mentioned in European seabass (*Dicentrarchus labrax*) (26), Yellow River carp (*Cyprinus carpio haematopterus*) (27), and grass carp (19). When assessing the quality characteristics of a muscle, the shear force is a crucial measure. At 90 days, tilapia fed FB60 and FB70 had shear forces that were noticeably higher than fish fed FB0. Enhanced dietary faba bean levels significantly increased muscle shear force, which is consistent with earlier research on grass carp that found that a partial substitution from soybean to faba beans had an impact on muscle shear force (19). The faba bean contain a variety of anti-nutritional factors such as total phenolics, tannins, and trypsin inhibitor activity. The tilapia muscle texture changes after dietary faba beans might be because of anti-nutritional factors in faba bean.

While the microstructure and structure concentrate on minor alterations in internal features and offer increasing data and further interpret the textural modifications caused by external settings, the textural properties typically focus more on physical information about the fish freshness (6). Fish muscle texture is closely related to myofibrillar structure that is much impacted by rearing situations (7). The current research pointed out that dietary faba bean not only significantly increased hardness, but also significantly reduced muscle fiber diameter and increased muscle density in tilapia. Some studies have also highlighted that textural features have a substantial favorable association with the muscle fiber density (28, 29). Both connections between the density of muscle fibers and muscle hardness showed a clear positive correlation (13, 30). Tilapia muscle fiber density rose at appropriate levels of dietary faba bean, showing that these levels promoted muscle fiber growth and that the rise in density may be linked to hyperplasia.

The muscle’s nutrient composition is a key measure for assessing fish meat quality, and the protein and amino acid contents and type can have an effect on value of nutrients, flavor and function of the fish muscle (9). In this study, the muscle ∑TAA content in tilapia fed FB60 was significantly higher than that of fed FB0, which may be the faba beans seeds are rich in protein and amino levels. When the hydrophobicity or acidity of the R group of the side chain of the amino acid is low, it exhibits an umami taste, whereas when the hydrophobicity is high, it exhibits a bitter taste. In the present study, the leucine and arginine contents in tilapia fed 60% faba bean were considerably higher in contrast to the control group, which may be the reason why faba bean transformed the amino acid content structure. Faba bean, with their higher levels of arginine, would supplement some of the ∑EAA compounds, thereby achieving a more balanced and desirable amino acid profile (31). Prior investigations have claimed that the activity and release of a number of hormones is stimulated by arginine, such as insulin-like growth factor (IGF)-1 and insulin, which stimulate growth, and protein metabolism (32), and leucine has been documented to stimulate postprandial free amino acid synthesis and IGF-1 signaling. The amino acid content was increased in fish fed faba beans, suggesting that faba beans might enhance the flavor of fish muscle and its nutritional value.

The lipid content in muscle is closely associated with flesh’s nutritional value, tenderness, and flavor (9), and present fatty acids in muscles of fish have an impact on the well-being of human (33). The total lipid content of the tilapia fed faba bean in the current study was lower in comparison to control group. The drop in crude lipid content of fish muscle is typically thought to promote friction among muscle bundles, increase muscle hardness, and lessen muscle sensitivity (34). Previous studies have also reported a reduction in muscle lipid content in rainbow trout (*Oncorhynchus mykiss*) (34), Yellow River carp (27) and beluga (*Huso huso*) (35) fed faba bean. This may be attributed to the fact that when some anti-nutritional factors in faba bean goes to a specific level, they influence the process of the transportation of lipids leading to a reduction in fat deposition concentration in the muscle of fish. The species and contents of fatty acids have a significant influence on the nutritional value of lipids. According to the World Health Organization, reducing the amount of saturated fatty acids and rising amount of unsaturated fatty acids in the human diet can improve nutrition and help prevent chronic diseases (36). Tissue fatty acid content of farmed fish has been reported to be greatly to be greatly influenced by dietary nutritional composition. The faba bean are rich fatty acid and have particularly high unsaturated fatty acid levels (31). In the current investigation, the total ∑SFA content reduced and ∑PUFA content enhanced, suggesting that dietary faba bean might enhance the health effects of fish muscle.

## 5. Conclusion

The outcomes of the current investigation revealed that dietary faba bean increased hardness, shear force and muscle fiber diameter in tilapia. These outcomes indicate that the smaller size of the fiber and increased fiber density played a major role in the better filet texture of tilapia. Faba bean also influenced muscle amino acid composition, lipids, saturated and unsaturated fatty acid contents. The current investigation effectively showed that dietary faba bean levels significantly affected texture, histology, and amino acid composition in tilapia over the 90-days feeding experiment. To better understand how faba beans regulate the quality of the tilapia muscle, more research should be done.

## Data availability statement

The raw data supporting the conclusions of this article will be made available by the authors, without undue reservation.

## Ethics statement

The animal study was reviewed and approved by Committee on Animal Research and Ethics of Zhongkai University of Agriculture and Engineering.

## Author contributions

QL was responsible for the conception and design of the experiments and writing the manuscript. YH was collected samples and performed the detection of nutrition index. XZ was organized the data and visualized part of the data. CZ was responsible for the visualization of figures and the revision of the manuscript. LL was provided supervision, validation, and funding support. All authors contributed to the manuscript and gave their approval for its submission.

## Funding

This work was supported by the Guangdong Province Commissioner for Rural Science and Technology in Towns and Village [grant number KTP20210236]; the Key Research and Development Program of Guangzhou City [grant number 202103000067]; and the College Student Innovation and Entrepreneurship Training Program [grant number S202211347069].

## Conflict of interest

The authors declare that the research was conducted in the absence of any commercial or financial relationships that could be construed as a potential conflict of interest.

## Publisher’s note

All claims expressed in this article are solely those of the authors and do not necessarily represent those of their affiliated organizations, or those of the publisher, the editors and the reviewers. Any product that may be evaluated in this article, or claim that may be made by its manufacturer, is not guaranteed or endorsed by the publisher.
